# Palmitate impairs circadian transcriptomics in muscle cells through histone modification of enhancers

**DOI:** 10.26508/lsa.202201598

**Published:** 2022-10-27

**Authors:** Nicolas J Pillon, Laura Sardón Puig, Ali Altıntaş, Prasad G Kamble, Salvador Casaní-Galdón, Brendan M Gabriel, Romain Barrès, Ana Conesa, Alexander V Chibalin, Erik Näslund, Anna Krook, Juleen R Zierath

**Affiliations:** 1 Department of Physiology and Pharmacology, Section of Integrative Physiology, Karolinska Institutet, Stockholm, Sweden; 2 Department of Molecular Medicine and Surgery, Section of Integrative Physiology, Karolinska Institutet, Stockholm, Sweden; 3 Novo Nordisk Foundation Center for Basic Metabolic Research, University of Copenhagen, Copenhagen, Denmark; 4 Biobam Bioinformatics S.L, Valencia, Spain; 5 Department of Microbiology and Cell Science, University of Florida, Gainesville, FL, USA; 6 Division of Surgery, Department of Clinical Sciences, Danderyd Hospital, Karolinska Institutet, Stockholm, Sweden

## Abstract

Obesity and elevated circulating lipids may impair metabolism by disrupting the molecular circadian clock. We tested the hypothesis that lipid overload may interact with the circadian clock and alter the rhythmicity of gene expression through epigenomic mechanisms in skeletal muscle. Palmitate reprogrammed the circadian transcriptome in myotubes without altering the rhythmic mRNA expression of core clock genes. Genes with enhanced cycling in response to palmitate were associated with post-translational modification of histones. The cycling of histone 3 lysine 27 acetylation (H3K27ac), a marker of active gene enhancers, was modified by palmitate treatment. Chromatin immunoprecipitation and sequencing confirmed that palmitate exposure altered the cycling of DNA regions associated with H3K27ac. The overlap between mRNA and DNA regions associated with H3K27ac and the pharmacological inhibition of histone acetyltransferases revealed novel cycling genes associated with lipid exposure of primary human myotubes. Palmitate exposure disrupts transcriptomic rhythmicity and modifies enhancers through changes in histone H3K27 acetylation in a circadian manner. Thus, histone acetylation is responsive to lipid overload and may redirect the circadian chromatin landscape, leading to the reprogramming of circadian genes and pathways involved in lipid biosynthesis in skeletal muscle.

## Abstract (precis)

The disruption of circadian rhythms because of lipid overload may lead to epigenomic changes that influence metabolism. Thus, a dietary or therapeutic modulation of lipid levels, a cornerstone in the treatment of metabolic disorders, may prevent circadian misalignment in peripheral tissues.

## Introduction

Obesity is characterized by increased circulating fatty acids and lipid accumulation in central and peripheral tissues, with associated metabolic disturbances and insulin resistance (Kelley et al, 1999). Obesity is also tightly linked to disrupted circadian rhythms—endogenous 24-h cycles that allow organisms to anticipate diurnal changes in physiology and behavior (Hsieh et al, 2010; Tahira et al, 2011; Vieira et al, 2014; Sardon Puig et al, 2020). Circadian misalignment increases circulating levels of glucose, free fatty acids, and triglycerides (Wefers et al, 2018) and adversely affects circulating leptin and ghrelin levels (Qian et al, 2019), which collectively can have deleterious consequences on whole-body glucose and energy homeostasis.

The disruption of the circadian rhythms in humans by sleep deprivation or shift work increases the risk of cardiometabolic diseases, including type 2 diabetes and obesity (van Drongelen et al, 2011; Rajaratnam et al, 2013; Dominguez et al, 2019). Similarly, simulated chronic jet lag in mouse models disturbs circadian rhythms and leads to leptin resistance and obesity (Kettner et al, 2015). Moreover, mice expressing a dysfunctional splice variant of the core circadian gene *Clock* are hyperphagic and develop obesity, with systemic alterations in glucose and energy homeostasis (Turek et al, 2005). Collectively, these results provide evidence to suggest that crosstalk exists between metabolic health, nutritional status, and circadian rhythms. Nevertheless, the relationship between biological clocks and metabolism is complex and bidirectional, and dietary interventions or metabolic diseases can disrupt circadian rhythms.

Circadian rhythms are controlled by transcriptional regulation and post-translational modifications (Young, 2018; Kim & Lazar, 2020). The core clock is composed of cell-autonomous transcription-translation feedback loops, comprised of a CLOCK:BMAL1 heterodimer that transcribes feedback repressors PER, CRY, and NR1D1 (Young, 2018; Kim & Lazar, 2020). The CLOCK:BMAL1 heterodimer can also be coupled to epigenomic mechanisms via histone modifiers, with CLOCK acting as a histone acetyltransferase (HAT), thereby altering gene expression through post-translational modifications (Naruse et al, 2004; Doi et al, 2006; Hirayama et al, 2007; Katada & Sassone-Corsi, 2010; Koike et al, 2012; Aguilar-Arnal et al, 2015). Intimate links between epigenetic regulation and the circadian clock exist that are likely to contribute to the plasticity of insulin-sensitive organs and metabolic control.

Circadian transcription is synergistically regulated by different environmental factors, including energetic states, levels of metabolites, and availability of fuel substrates (Lamia et al, 2009; Eckel-Mahan et al, 2012; Vollmers et al, 2012; Adamovich et al, 2017; Krishnaiah et al, 2017; Greco et al, 2020; Manella et al, 2020). Calorie restriction enhances the amplitude of clock genes and results in an accumulation of histone acetylation (H3K9/K14 and H3K27) at circadian hepatic promoters (Sato et al, 2017). Long-term high-fat diets alter the core clock machinery and clock-controlled genes in mouse tissues (Feng et al, 2011; Tognini et al, 2017). Thus, dietary factors reprogram the circadian clock through epigenetic processes, leading to the circadian dysregulation of metabolic homeostasis and the onset of metabolic diseases.

Men and women with obesity present altered mRNA expression of core clock genes in skeletal muscle, blood cells, and visceral adipose tissue (Tahira et al, 2011; Vieira et al, 2014; Sardon Puig et al, 2020). Moreover, the altered core clock gene expression in skeletal muscle from men and women with obesity correlates with circulating fatty acid levels (Sardon Puig et al, 2020). In mouse model, diet-induced obesity reprograms circadian gene regulation through the rewiring of lipid metabolic pathways (Eckel-Mahan et al, 2013; Guan et al, 2018). Given the link between clock gene expression, lipid metabolism, and metabolic disease, we tested the hypothesis that saturated fatty acids may alter the rhythmicity of gene expression in skeletal muscle. We discovered that the saturated fatty acid palmitate regulates enhancer activity through the regulation of histone H3 lysine K27 acetylation and alters skeletal muscle circadian transcriptomics.

## Results

### Palmitate treatment alters circadian oscillations in primary human myotubes

**Figure 1.**
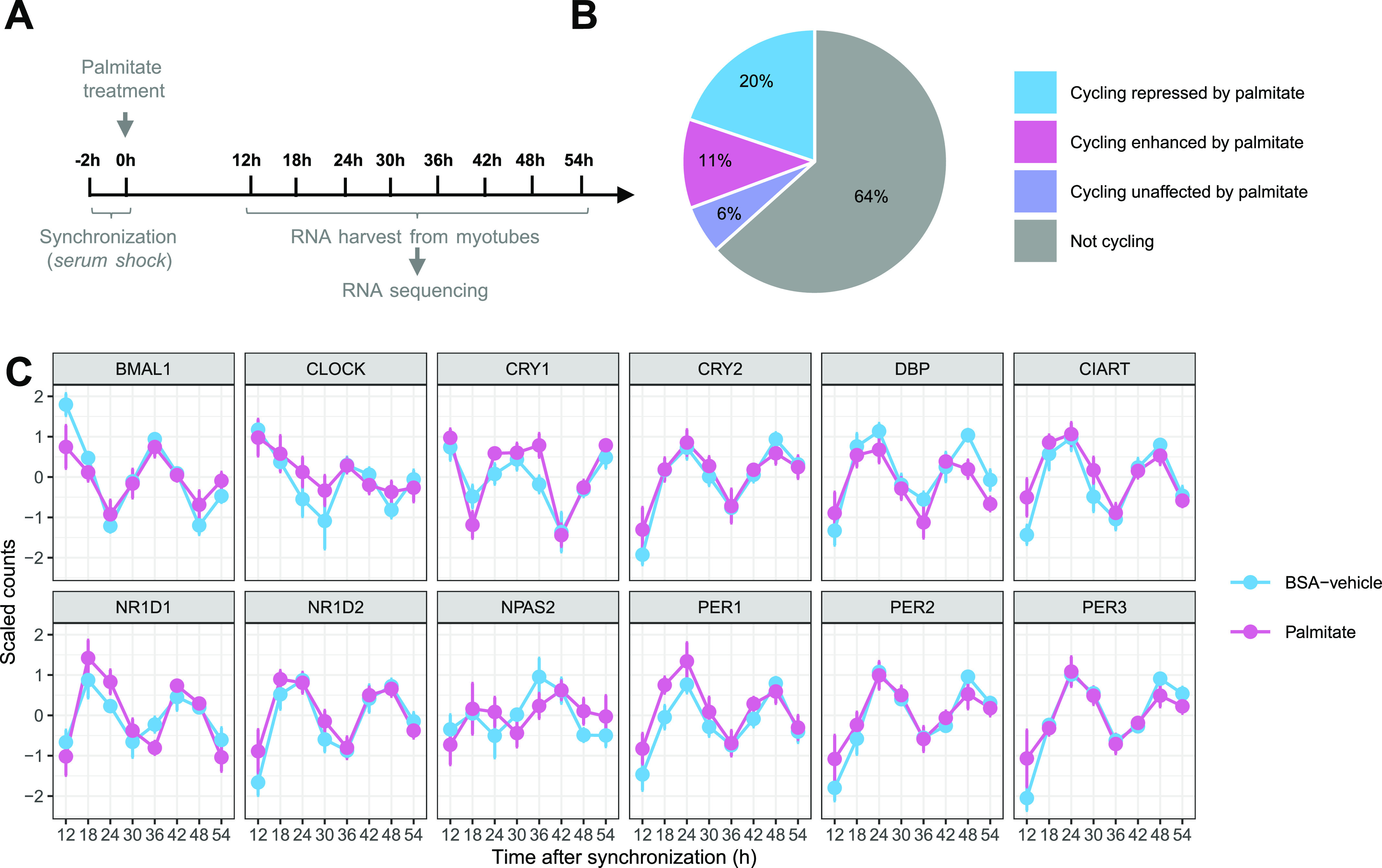
Palmitate alters the pattern of rhythmic transcripts. **(A)** Graphic representation of data collection and RNA-seq of synchronized primary human skeletal muscle myotubes (n = 7) treated with palmitate (0.4 mM) or BSA-vehicle. **(B)** Proportion of rhythmic genes (RAIN analysis, FDR < 0.1) cycling only in the BSA condition (repressed by palmitate), only in the palmitate condition (enhanced by palmitate), or in both conditions (unaffected by palmitate). **(C)** Core clock gene expression in synchronized myotubes. Data are the mean ± SE, n = 7.

Primary skeletal muscle cells were synchronized, treated with palmitate or BSA-vehicle, and harvested every 6 h (Fig 1A). The RAIN algorithm was used to determine rhythmic oscillations of genes and identified 37% of all transcripts as cycling (Fig 1B). Genes cycling in the control condition, but not after palmitate treatment, were considered repressed by palmitate and represented 20% of all transcripts and >50% of cycling transcripts (Fig 1B). Conversely, genes not cycling in the control condition, but cycling after palmitate treatment, were induced by palmitate and represented 11% of all transcripts. Genes cycling in both BSA-vehicle– and palmitate-treated conditions were considered unaffected by palmitate and only represented 6% of all detected transcripts (Fig 1B). Core clock genes, including *BMAL1*, *CIART*, *DBP*, *CRY1*, *CRY2*, *NR1D1*, *NR1D2*, *PER1*, *PER2*, and *PER3*, were unaffected by palmitate (Fig 1C). Only *CLOCK* and its paralog *NPAS2* were rhythmic in BSA-vehicle–treated, but not in palmitate-treated, myotubes (Fig 1C). Thus, palmitate influenced the number of rhythmic genes, with minimal effect on the mRNA expression of core clock machinery components.

**Figure 2.**
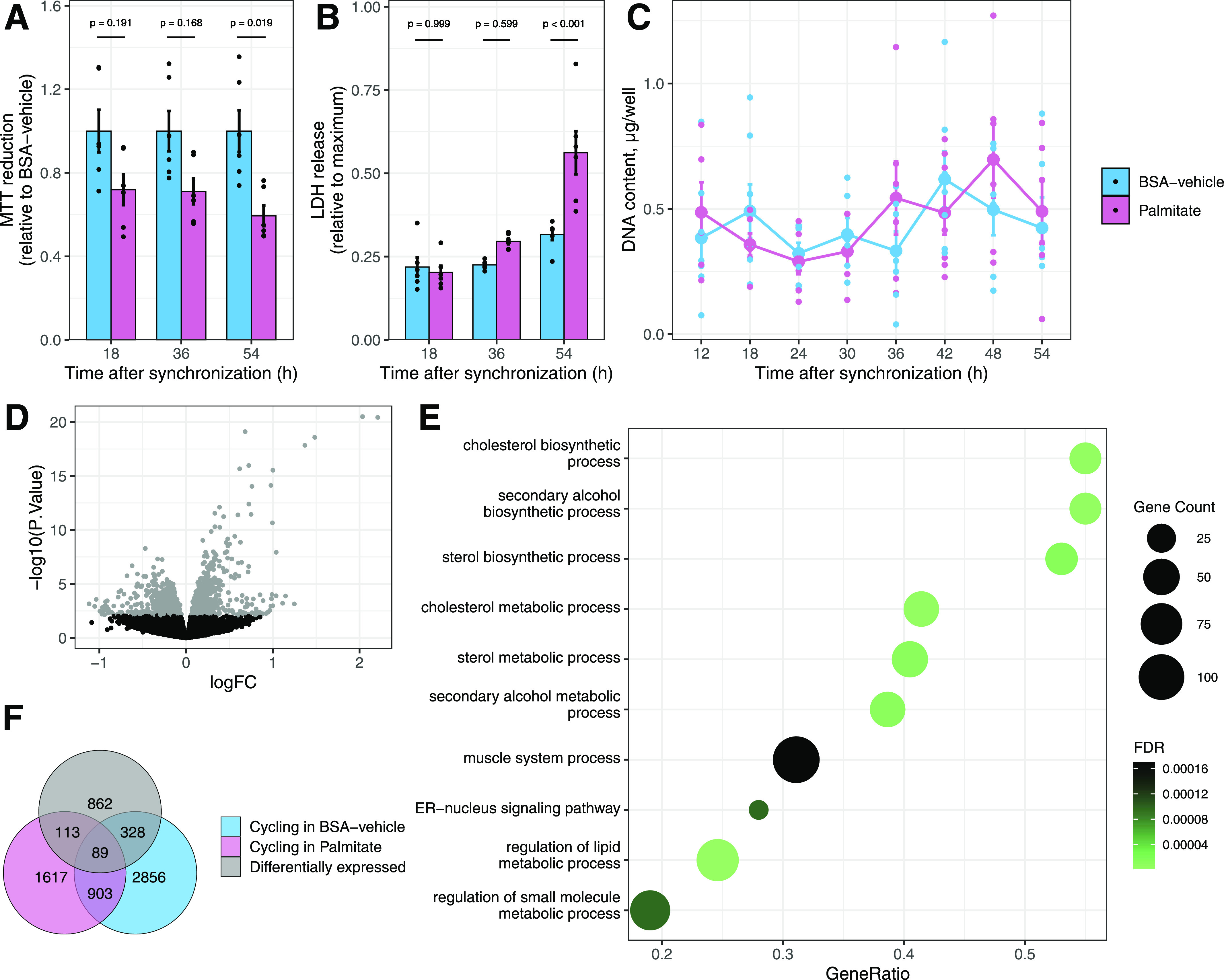
Palmitate alters the pattern of rhythmic transcripts. **(A)** MTT assay to estimate metabolic activity in response to palmitate. Data are the mean ± SE, n = 6. **(B)** Cytotoxicity of palmitate estimated by the release of LDH in the supernatant of cultured cells. Data are the mean ± SE, n = 6. **(C)** DNA content per well over the 54 h of treatment with palmitate. Data are the mean ± SE, n = 7. **(D)** Differential expression analysis of rhythmic genes in palmitate-treated myotubes (limma, FDR < 0.1). **(E)** Gene set enrichment analysis of gene ontology biological processes for differentially expressed genes. **(F)** Overlap between genes differentially regulated by palmitate (limma, FDR < 0.1) and genes with cycling repressed or enhanced by palmitate (RAIN, FDR < 0.1).

In human primary skeletal muscle cells, the palmitate (0.4 mM) exposure reduced NAD(P)H oxidoreductase activity (Fig 2A), demonstrating that palmitate induced metabolic stress early, in response to treatment. Cell death was not detectable at these early time points, and the release of LDH into the extracellular milieu was triggered only after 54 h of palmitate exposure (Fig 2B). Accordingly, DNA content per well remained constant throughout the experiment (Fig 2C). Collectively, these control experiments provide evidence that cytotoxicity is unlikely to account for the differences in palmitate-induced transcriptional rhythmicity.

Changes in gene expression after exposure to saturated fatty acids have been extensively described in multiple model systems (Georgiadi & Kersten, 2012). To test whether genes where cycling was affected by palmitate overlapped with genes where total mRNA abundance is typically changed in response to fatty acids, we performed a differential expression analysis after blocking for the effect of time. Changes in total mRNA abundance after palmitate exposure were observed in 9% of all transcripts (Fig 2D). Palmitate-responsive genes were associated with gene ontology pathways related to lipid metabolism (Fig 2E). Little overlap was observed between genes differentially expressed and genes with dysregulated cycling (Fig 2F), suggesting that an alteration in circadian rhythmicity by palmitate treatment involves mechanisms separate from the canonical lipid metabolic pathways activated by fatty acids.

### Distinct rhythmic gene ontologies in palmitate-treated human myotubes

**Figure 3.**
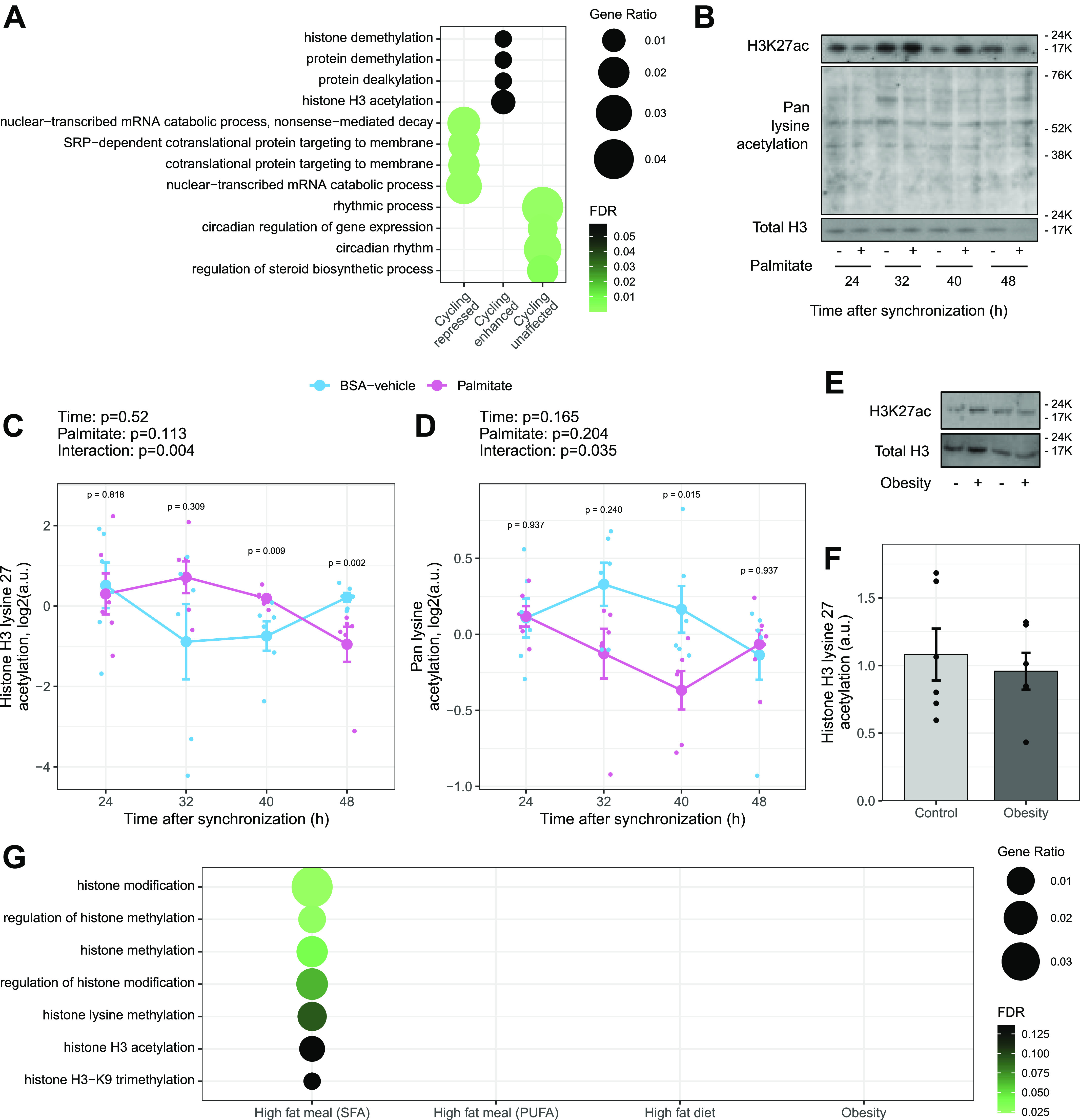
Palmitate alters the circadian acetylation of histone H3 on lysine 27. **(A)** Gene ontology overrepresentation analysis of biological processes on genes cycling only in BSA-vehicle, only in palmitate, or in both conditions. **(B)** Immunoblots of histone 3 acetylation from BSA-vehicle– and palmitate-treated myotubes every 8 h after synchronization. **(C, D)** Quantification of lysine 27 acetylation and pan-lysine acetylation. Results are the mean ± SEM, n = 6, paired two-way ANOVA (time, palmitate), followed by the pairwise Wilcoxon comparisons. **(E, F)** Relative abundance of total histone H3 and histone H3K27ac in skeletal muscle biopsies obtained from men with normal weight (n = 6) or obesity (n = 6). Results are the mean ± SEM, n = 6. **(G)** Enrichment of pathways related to histone modification in skeletal muscle biopsies collected before and after a saturated fat meal (SFA), a polyunsaturated fat meal (PUFA), at the fasted state after 3 d of a high-fat diet, or at the fasted state in men with obesity versus normal weight. Data from the publicly available datasets were processed as described in the Materials and Methods section.

Gene ontology overrepresentation analysis for biological processes on the cycling genes unaffected by palmitate showed an enrichment for rhythmic processes and circadian regulation (Fig 3A). Most core clock genes belong to these pathways, confirming the absence of effect of palmitate on the mRNA cycling of the core clock and suggesting that palmitate reprograms circadian transcriptome independently of the core clock machinery. Genes where cycling was repressed by palmitate were associated with pathways involved in transcription and protein targeting to membrane (Fig 3A). Genes where cycling was induced in response to palmitate were annotated to pathways related to post-translational modifications of histones (Fig 3A).

To explore a potential mechanism for the altered temporal regulation of transcription, we investigated whether palmitate treatment altered cyclic histone modifications in synchronized primary human myotubes. The relative abundance of total histone H3, total acetylated lysine, and the marker of active enhancers, histone H3 lysine 27 (H3K27ac), was assessed over a 48-h period in BSA-vehicle– and palmitate-treated myotubes (Fig 3B). Histone H3 protein abundance was unaffected by time or palmitate treatment, whereas the acetylation of histone 3 on lysine 27 (H3K27ac) was affected by palmitate in a time-dependent manner (RAIN, *P* < 0.05; Fig 3C). Total lysine acetylation exhibited similar palmitate-affected rhythms (RAIN, *P* < 0.05), but this regulation was opposite to that of H3K27ac (Fig 3D), suggesting palmitate exposure led to a specific enrichment in histone H3K27ac over total cellular lysine acetylation.

Dysregulated lipid homeostasis is associated with metabolic diseases. Individuals with obesity exhibit elevated levels of circulating free fatty acids concomitant with disturbances in circadian rhythmicity (Sardon Puig et al, 2020). To test whether obesity directly affects the acetylation of histones in skeletal muscle, the abundance of H3K27ac was measured in *vastus lateralis* biopsies from men with obesity versus normal weight (Fig 3E and F). We found that the acetylation of H3K27 was unaffected by obesity in biopsies obtained in the morning after an overnight fast. In publicly available datasets, gene ontology pathways related to histone modifications were enriched in skeletal muscle biopsies obtained from participants before and after a meal rich in saturated fat (Fig 3G). Conversely, pathways related to histone modifications were not significantly enriched in skeletal muscle obtained from participants after a meal rich in polyunsaturated fatty acids. In biopsies collected from fasted individuals after 3 d of high-fat feeding or from individuals with obesity versus normal weight, pathways related to histone modifications were not significantly enriched (Fig 3G). Collectively, these data in human skeletal muscle provide indirect evidence that changes in histone acetylation may be a consequence of acute fatty acid exposure rather than chronic obesity.

### Palmitate attenuates the rhythmic behavior of H3K27-acetylated regions

**Figure 4.**
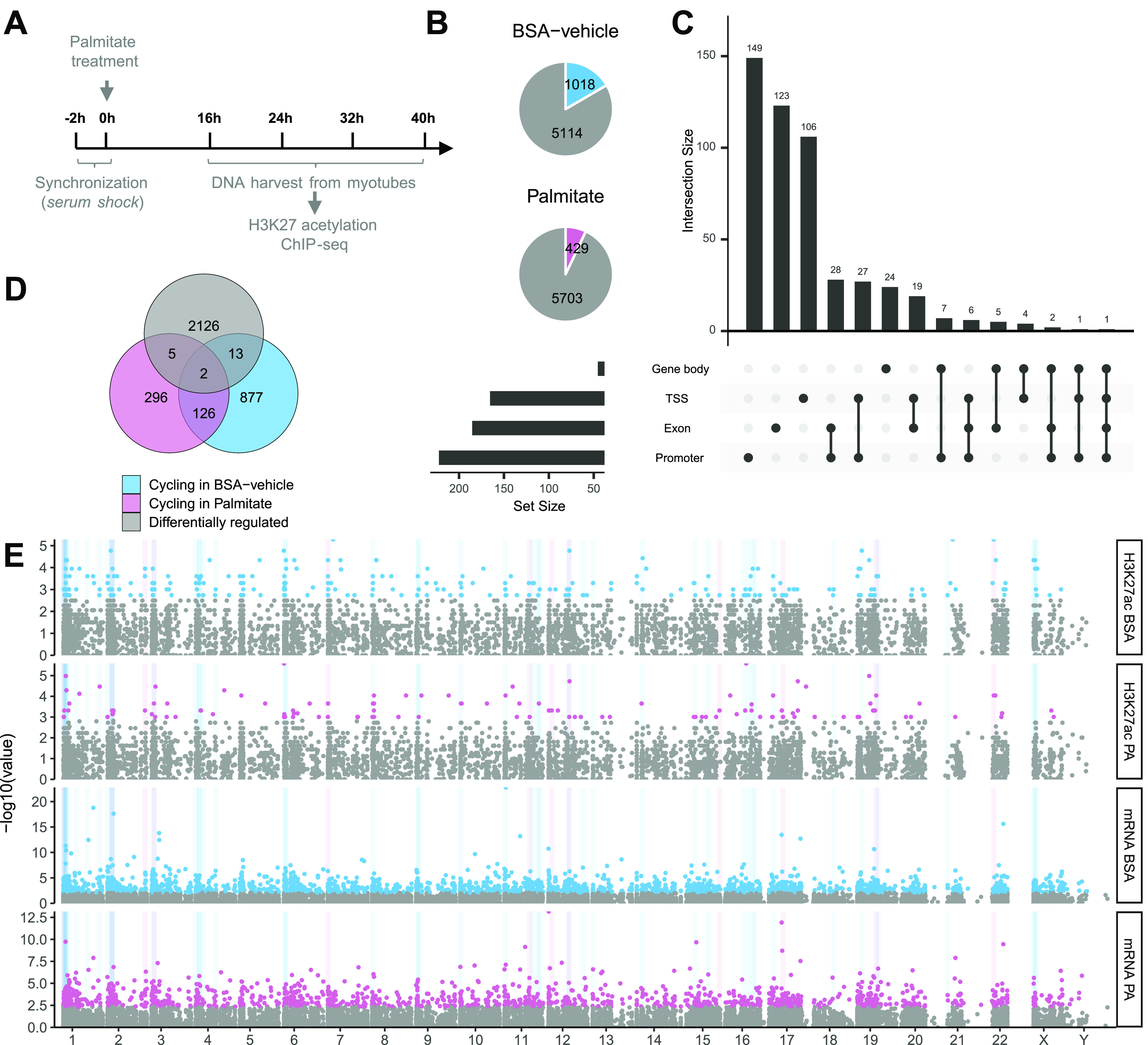
Palmitate treatment reduces the cycling of H3K27-acetylated regions. H3K27ac ChIP-seq of synchronized primary human skeletal muscle myotubes (n = 4) treated with palmitate (0.4 mM) or BSA-vehicle. **(A)** Graphic representation of data collection. **(B)** Proportion of rhythmic regions identified (RAIN analysis, FDR < 0.1). **(C)** Location of acetylated regions in gene body, transcription starting site, exons, or promoter regions of their associated genes. **(D)** Differential acetylation analysis compared with regions cycling in BSA-vehicle– or palmitate-treated myotubes. **(E)** Manhattan plot of cycling genes and regions associated with H3K27ac. From top to bottom: cycling H3K27ac in BSA-vehicle, cycling H3K27ac in palmitate, mRNA cycling in BSA-vehicle, and mRNA cycling in palmitate. Grey areas highlight regions of the genome with cycling at both mRNA and H3K27ac in either BSA-vehicle or palmitate.

Genome-wide analysis of histone H3K27ac was performed by chromatin immunoprecipitation (ChIP) and sequencing of synchronized primary human muscle cells treated with palmitate or BSA-vehicle (Fig 4A). We identified more than 127,000 regions with acetylated peaks that were distributed throughout the genome. From these, 6,132 regions were annotated to a gene and used for further analysis. In BSA-vehicle–treated myotubes, we identified 1,018 rhythmic regions, compared with 429 rhythmic regions in palmitate-treated myotubes (Fig 4B). Acetylation regions were not only predominant in promoter and transcription starting sites but also present in exons and gene bodies (Fig 4C).

Like the changes observed in mRNA, regions differentially acetylated after palmitate treatment were distinct from the regions with changes in cycling (Fig 4D), suggesting different mechanisms involved in the regulation of cycling compared with simple mRNA transcription. These results suggest that the palmitate-induced acetylation of specific H3K27ac enhancer regions is regulated in a circadian manner, and this may be responsible for changes in the rhythmicity of gene expression in response to fatty acids.

Our analysis revealed that palmitate affects the circadian rhythm of many genes, independent of changes in differential expression. Furthermore, we provide evidence that palmitate regulates the acetylation of H3 on lysine 27, a hallmark of enhancers. Thus, we next identified genes with coincident rhythmic mRNA profiles and enhancer regions. Manhattan plots demonstrated that cycling genes and enhancer regions were distributed across the entire genome, with no enrichment localized to any specific chromosome (Fig 4E).

### Changes in rhythmic acetylation of enhancers affect palmitate-responsive genes

**Figure 5.**
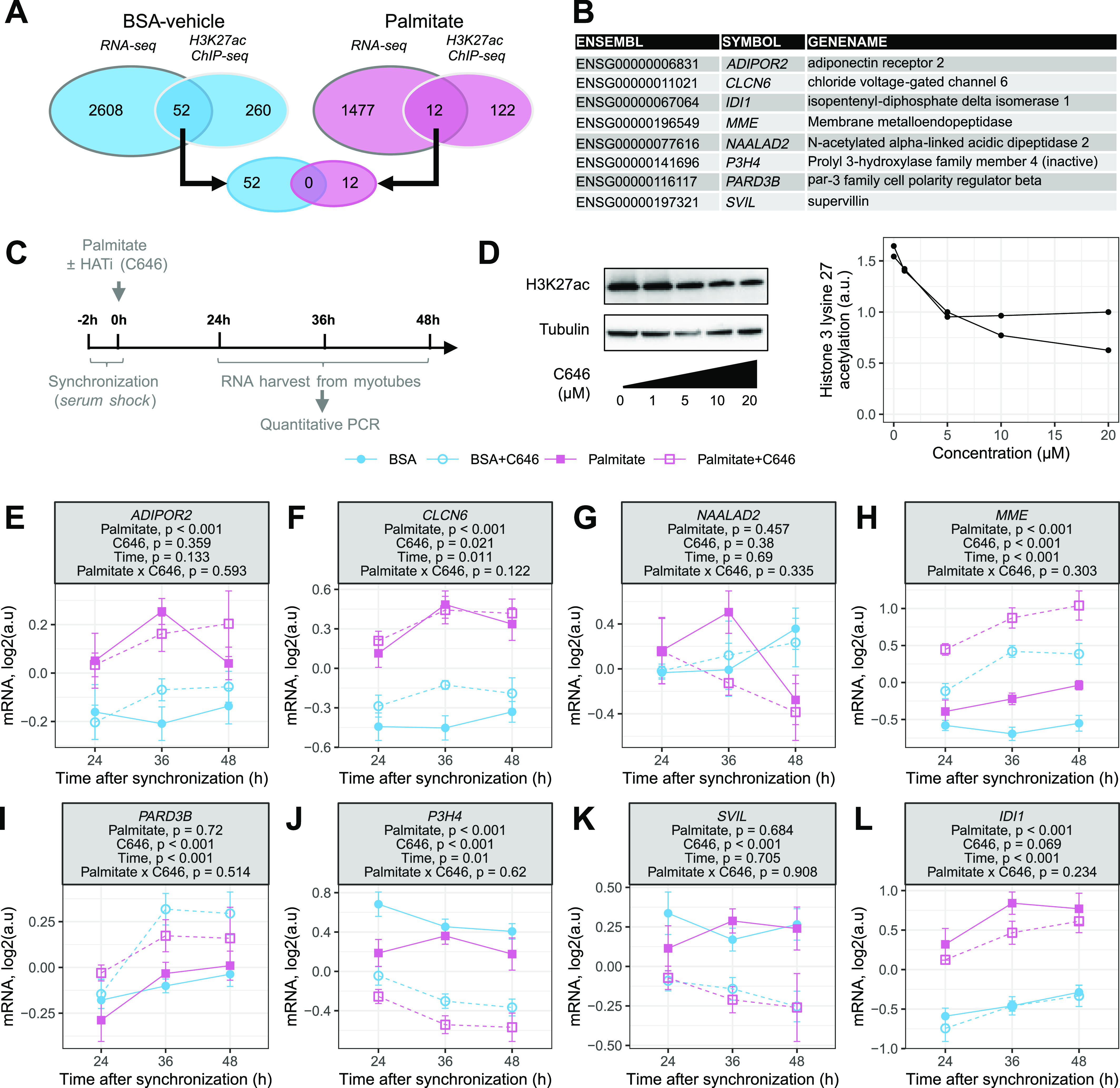
Changes in enhancer rhythmic acetylation and rhythmic transcriptomics in myotubes. **(A)** Overlap between rhythmic genes (RNA-seq, FDR < 0.05) and genes associated with rhythmic regions (H3K27ac ChIP-seq, FDR < 0.05) in BSA-vehicle–treated (blue) and palmitate-treated (pink) myotubes. **(B)** Genes with both cycling mRNA and H3K27ac-associated regions and differentially expressed in myotubes. **(C)** Graphic representation of data collection. **(D)** Efficiency of inhibition of histone 3 lysine 27 acetylation by the histone acetyltransferase inhibitor C646. Representative blots and quantification (n = 2). **(E, F, G, H, I, J, K, L)** mRNA expression of cycling genes identified as regulated by palmitate in panel (B). Data are the mean ± SEM, n = 8; results of a three-way ANOVA (palmitate, C646, time) are presented under the gene names.

We identified 52 genes in BSA-vehicle–treated and 12 genes in palmitate-treated myotubes with cycling at both the mRNA and the H3K27ac regions (Fig 5A). Of those, only eight genes were also differentially expressed in myotubes (Fig 5B), confirming that changes in mRNA rhythms can occur independently of changes in total mRNA abundance.

HATs have a significant role in the acetylation of histones and could therefore be responsible for the palmitate-induced changes in rhythmic transcription. Synchronized myotubes were exposed to the HAT inhibitor C646 throughout the palmitate treatment (Fig 5C) to inhibit the acetylation of histone H3K27 (Fig 5D). The mRNA expression of genes was assessed by quantitative PCR. HAT inhibition differentially altered the mRNA expression of several genes (Fig 5E–L). *ADIPOR2*, *CLCN6*, and *NAALAD2* were nominally affected by HAT inhibition (Fig 5E–G). The overall mRNA expression of *MME* and *PARD3B* was similarly elevated by C646 in both the BSA-vehicle– and the palmitate-treated myotubes (Fig 5H and I). Conversely, mRNA levels of *P3H4* and *SVIL* were decreased by HAT inhibition, without any significant difference between BSA-vehicle– and palmitate-treated myotubes (Fig 5J and K). HAT inhibition slightly reduced the effect of palmitate on the mRNA expression of *IDI1* (Fig 5L), with the strongest effect observed 36 h after synchronization. Collectively, these changes in mRNA demonstrate that HAT inhibition has wide, diverse, and selective effects on the expression of genes, suggesting complex interactions between histone acetylation and the response to fatty acids.

### Inhibition of histone acetylation prevents palmitate-induced activation of lipid biosynthesis pathways

**Figure 6.**
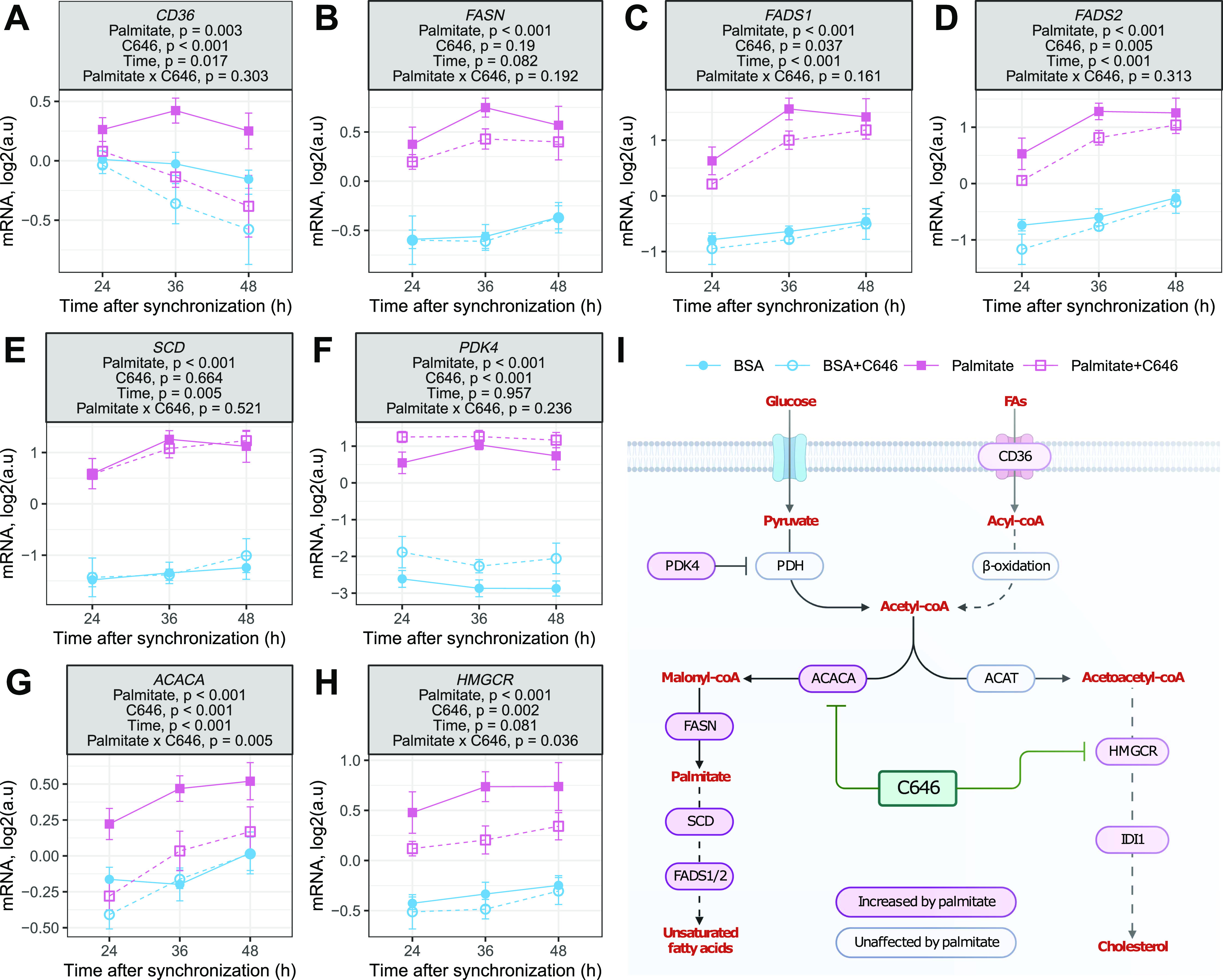
Inhibition of histone acetyltransferases affects the mRNA expression of palmitate-responsive genes. **(A, B, C, D, E, F, G, H)** mRNA expression of genes involved in lipid uptake and synthesis. Data are the mean ± SEM, n = 8; results of a three-way ANOVA (palmitate, C646, time) are presented under the gene names. **(I)** Schematic representation of genes affected by palmitate and the HAT inhibitor C646. Created with BioRender.com.

PARD3B and P3H4 are associated with body fat, and CLCN6 is associated with low-density lipoprotein cholesterol in GWAS (Buniello et al, 2019). ADIPOR2 signals to PPARA activity to increase fatty acid oxidation, whereas IDI1 is involved in cholesterol synthesis. The overlap between our results generated by RNA-seq and H3K27ac-ChIP-seq suggests that HAT inhibition may regulate lipid-related pathways in response to fatty acid exposure. Palmitate exposure increased the mRNA expression of multiple genes involved in lipid biosynthetic pathways, including lipid transporter (*CD36*; Fig 6A), fatty acid synthase (*FASN*; Fig 6B), fatty acid desaturases (*FADS1/2*; Fig 6C and D), and stearoyl-CoA desaturase (*SCD*; Fig 6E). Palmitate also increased the mRNA expression of pyruvate dehydrogenase kinase 4 (*PDK4*; Fig 6F). However, HAT inhibition prevented the effect of palmitate on the mRNA expression of only two enzymes, namely, lipid acetyl-CoA carboxylase (*ACACA*; Fig 6G) and 3-hydroxy-3-methylglutaryl-CoA reductase (*HGMCR*; Fig 6H). Because these enzymes are rate-limiting for lipid and cholesterol synthesis pathways, respectively, this suggests that the palmitate-induced acetylation of histones could have major effects on lipid biosynthesis (Fig 6I).

## Discussion

Acute saturated fatty acid exposure disrupts rhythmic gene expression in skeletal muscle (Sardon Puig et al, 2020), consistent with fatty acid–induced clock disturbances in liver and adipose tissues (Vieira et al, 2014; Tal et al, 2019b). Here, we show that the saturated fatty acid palmitate triggers transcriptomic and epigenomic changes in skeletal muscle cells that involve alterations in the histone acetylation and activation of enhancers, leading to the disruption of circadian gene expression. Palmitate treatment repressed the rhythmicity of genes involved in protein translation and transport, and enhanced the rhythmicity of genes involved in histone modifications. Palmitate changed the rhythmicity of enhancers via the modification of histone H3K27ac, leading to changes in the circadian rhythmicity of selective subsets of genes. Our findings provide insight into mechanisms by which saturated fatty acids alter metabolic homeostasis via circadian misalignment.

Long-term palmitate exposure has cytotoxic effects and induces insulin resistance (Makinen et al, 2017; Pillon et al, 2018). These disturbances coincide with changes in transcription and altered lipid metabolism (Dimopoulos et al, 2006; Hall et al, 2014; Tal et al, 2019b). Increased fatty acid levels promote lipid oxidation, which is the primary contributor to the global acetyl-CoA pool (McDonnell et al, 2016). Increased acetyl-CoA levels promote histone acetylation (McDonnell et al, 2016), one of the most prominent marks leading to the activation of gene expression (Kim & Lazar, 2020). Here, we demonstrate that in myotubes, palmitate treatment decreased total lysine acetylation, concomitant with changes in rhythmicity and increased histone H3K27ac. Thus, acute exposure to saturated fatty acids imparts post-transcriptional modifications to histone proteins, supporting emerging evidence for lipid-induced epigenomic control of genes important for homeostatic/lipotoxic programs (Yoon et al, 2021).

HATs and histone deacetylases are enzymes that regulate the lysine acetylation of proteins, in particular, histones. HATs are linked to the circadian control of metabolism (Kim & Lazar, 2020). We found that HAT inhibition alters palmitate-induced changes in the rhythmic expression of genes related to lipid biogenesis, indicating that the modification of histone acetylation by saturated fatty acids affects the transcriptomic regulation of selective subsets of genes. Several metabolic sensors act as intermediates to couple circadian rhythms and metabolism. Sirtuins (SIRTs) are NAD^+^-dependent histone deacetylases that sense cellular energy metabolism and whose activity follows a circadian pattern (Nakahata et al, 2008). Components of the core clock machinery, CLOCK:BMAL1, coexist with SIRT1 in a chromatin regulatory complex (Nakahata et al, 2008). CLOCK can act as a HAT, with specificity for histones H3 and H4 (Doi et al, 2006), and interact with SIRT1 to regulate the acetylation and deacetylation of BMAL1, respectively, which is essential for the circadian regulation of gene expression (Hirayama et al, 2007). In hepatocytes and adipocytes, palmitate exposure disrupts circadian gene oscillations by preventing BMAL1 deacetylation and activation and interfering with the CLOCK:BMAL1 interaction (Tong et al, 2015; Tal et al, 2019a, 2019b). Thus, palmitate may alter NAD^+^ levels, SIRT1 activity, and CLOCK:BMAL1 action, which may consequently alter histone H3K27ac. Fluctuations of NAD^+^ levels are linked to peripheral clocks (Nakahata et al, 2009; Ramsey et al, 2009; Peek et al, 2013). The extent to which other metabolites and co-factors fluctuate throughout the day to maintain metabolic homeostasis is an emerging topic of research. Nutrient overload associated with high-fat feeding alters tissue-specific metabolomic profiles and leads to circadian misalignment (Dyar et al, 2018). Exercise and nutritional state at different times of the day also influence tissue-specific metabolomic profiles and enrichment of H3K9ac and H3K27ac target genes within myotubes, suggesting that epigenetic modifications of chromatin are influenced by energetic states and fuel substrates (Sato et al, 2019). The nature of the palmitate-induced intercellular metabolite that governs the epigenetic control of the clock machinery requires further interrogation. Nevertheless, our results have physiological implications by linking an oversupply of nutrients in the form of saturated fatty acids to the circadian machinery and the control of metabolism.

We observed circadian oscillations in the levels of histone H3K27ac in primary human muscle cells, supporting the notion that histone modifications are under circadian regulation (Feng et al, 2011; Koike et al, 2012; Sato et al, 2017; Kim et al, 2018). Palmitate treatment altered global H3K27ac levels, consistent with evidence that palmitate acetylates enhancer regions regulating lipid metabolism in skeletal muscle and liver cells (Nammo et al, 2018; Williams et al, 2020). Changes in histone H3 acetylation are an underlying mechanism regulating rhythmic transcriptional activity in mouse liver (Etchegaray et al, 2003). However, histone acetylation and specifically histone H3K27ac are likely accompanied by parallel changes in other covalent modifications that also regulate transcription (Orphanides & Reinberg, 2002). Changes in other histone marks, DNA methylation, mRNA stability, and/or post-transcriptional RNA processing, may also contribute to the transcript oscillations, even in the absence of enhancer regulation.

Our finding that palmitate treatment increased H3K27ac in cultured myotubes suggests that an acute elevation in circulating lipids may be a contributing factor to this histone modification. Changes in histone H3K27ac in skeletal muscle have been described in the context of aging, with an increased expression of genes regulating extracellular matrix structure and organizations (Zhou et al, 2019). We found histone H3K27ac was associated with changes in genes involved in fatty acid metabolism, consistent with earlier reports in the pancreas and colon of diet-induced obese mice (Li et al, 2014; Nammo et al, 2018). Conversely, histone H3K27ac was unaltered in skeletal muscle of men with obesity as compared to normal weight. Because the biopsies were taken in the morning, we cannot exclude the possibility that diurnal signaling of histone H3K27ac may differ between the groups. The biopsies were also taken in fasted individuals, and given that histone acetylation is transiently induced by fatty acids, our results may differ in fed individuals or in those after a high-fat meal. Nevertheless, our data suggest that lipid-induced changes, rather than obesity per se, may directly contribute to the metabolic dysregulation of histone H3K27ac observed in skeletal muscle.

In summary, the saturated fatty acid palmitate disrupts circadian transcriptomics in primary human myotubes. Our results provide a link between nutrient overload, disruptions of circadian rhythms, and metabolic pathways. Increased histone H3K27ac in palmitate-treated primary human myotubes suggests a specific role for this epigenetic mark in the transcriptional changes that occur in peripheral tissues in response to lipid overload. The disruption of circadian rhythms in skeletal muscle because of lipid overload may lead to epigenomic changes that influence metabolism. Thus, a dietary or therapeutic modulation of lipid levels, a cornerstone in the treatment of metabolic disorders, may prevent circadian misalignment in peripheral tissues.

## Materials and Methods

### Subjects

*V. lateralis* muscle biopsies were obtained from seven healthy men to establish primary skeletal muscle cell cultures to determine the effects of palmitate on circadian transcriptomics, histone H3 protein abundance, and histone H3 lysine 27 acetylation (H3K27ac). *V. lateralis* muscle biopsies were obtained from a cohort of men with normal weight (n = 6) or obesity (n = 6), and a portion of the biopsy was processed for immunoblot analysis of H3 protein abundance and H3K27ac. Biopsies were collected in the morning after an overnight fast. Clinical characteristics of men with normal weight or obesity are presented in Table 1. Studies were approved by the regional ethics committee of Stockholm and conducted in accordance with the Declaration of Helsinki (2012/1955-31/1, 2013/647-31/3, 2012/1047-31/2, and 2016/355-31/4). Participants gave informed consent before enrolment.

**Table 1.** Clinical characteristics of the study participants.

	Normal weight (men [n = 6])	Obesity (men [n = 6])
Age (yr)	53 ± 3	48 ± 2
Body weight (kg)	79.8 ± 2.4	103.3 ± 1.5*
BMI (kg/m^2^)	24.2 ± 0.2	31.9 ± 0.6*
fP-glucose (mmol/l)	5.4 ± 0.1	5.5 ± 0.3
fP-insulin (pmol/l)	39.0 ± 6.5	116.3 ± 24.5*
HOMA-IR	1.4 ± 0.2	4.4 ± 1.2*
HbA1c (mmol/mol)	35.7 ± 1.4	33.0 ± 2.7
fP-cholesterol (mmol/l)		
Total	4.9 ± 0.2	5.2 ± 0.4
LDL	3.3 ± 0.2	2.9 ± 0.5
HDL	1.3 ± 0.1	1.6 ± 0.3
fP-triglycerides (mmol/l)	0.9 ± 0.1	1.8 ± 0.3*

Results are the mean ± SEM for normal-weight men and men with obesity. Differences between normal-weight men and men with obesity were determined using an unpaired *t* test. *P < 0.05 versus normal weight.

### Primary human skeletal muscle cell cultures

Primary myoblasts were grown in DMEM/F12+GlutaMAX with 16% FBS and 1% Antibiotic-Antimycotic. Cells were regularly tested for mycoplasma contamination by PCR. At 80% confluence, myoblasts were differentiated into myotubes by culturing in a fusion medium consisting of 76% DMEM and GlutaMAX with 25 mM glucose, 20% M199 (5.5 mM), 2% Hepes, and 1% Antibiotic-Antimycotic (100×), with 0.03 μg/ml zinc sulfate and 1.4 mg/ml vitamin B12. Apo-transferrin (100 μg/ml) and insulin (0.286 IU/ml) were added to the fusion medium. After 4–5 d, the medium was switched to the same medium without apo-transferrin or insulin, with 2% FBS (post-fusion media), and the cultures were continued for 3–5 d.

### Palmitate treatment

Palmitate stock solution (200 mM) (C16, P9767; Sigma-Aldrich) was prepared in 50% ethanol and then diluted 25 times in a 10.5% BSA solution. BSA (A8806; Sigma-Aldrich) in serum-free essential α-medium was used as a carrier and control. Myotubes were incubated in 5.5 mM glucose media for 22 h before the experiments. Myotubes were synchronized by serum shock (50% FBS, 2 h), washed with PBS, and incubated in a 5.5 mM glucose medium containing palmitate (0.4 mM) or BSA (vehicle). Cultures were collected every 6 h for mRNA analysis and every 8 h for DNA and immunoblot analyses, starting from 12 to 54 h after synchronization.

### Cell viability

The viability of the cells was tested after synchronization and exposure to palmitate. NAD(P)H oxidoreductase activity was measured using the MTT assay. Skeletal muscle cells were exposed to palmitate and incubated with 0.5 mg/ml thiazolyl blue tetrazolium bromide diluted in a culture medium. After 1.5 h, the medium was removed, and formazan crystals were dissolved with DMSO. Absorbance was read at 550 using a microplate reader. Lactate dehydrogenase activity in the supernatant from the cells was measured using the Cytotoxicity Detection Kit (Roche) according to the manufacturer’s instructions. LDH activity was normalized to the total LDH activity obtained after permeabilizing cells with 0.1% Triton X-100. DNA was extracted using the E.Z.N.A. tissue extraction kit following the manufacturer’s instructions, and DNA concentration was measured with a NanoDrop 1000 spectrophotometer (Thermo Fisher Scientific).

### Immunoblot analysis

Myotube cultures were lysed in ice-cold buffer A (1% protease inhibitor cocktail, 137 mmol/l NaCl, 2.7 mM KCl, 1 mM MgCl_2_, 5 mM Na_4_P_2_O_7_, 0.5 mM Na_3_VO_4_, 1% Triton X-100, 10% glycerol, 20 mM Tris, 10 mM NaF, 1 mM EDTA, and 0.2 mM phenylmethylsulfonyl fluoride, pH 7.8), followed by end-over-end rotation for 60 min (4°C) and centrifugation at 12,000*g* for 15 min (4°C). Skeletal muscle biopsies were pulverized in liquid nitrogen and lysed in homogenization buffer A supplemented with 0.5% of NP-40 and 0.02% of SDS, followed by end-over-end rotation for 60 min (4°C) and centrifugation at 3,000*g* for 10 min (4°C). Protein concentration was determined using a Pierce BCA protein assay kit (#23225; Thermo Fisher Scientific). Samples were prepared for SDS–PAGE with Laemmli buffer, separated on Criterion XT Bis-Tris 4–12% gradient gels (Bio-Rad), and transferred to PVDF membranes (Merck). Ponceau staining was performed, and the results were normalized to the total amount of protein per lane. Western blot was performed using primary antibodies (1:1,000 concentration) in TBS containing 0.1% BSA and 0.1% NaN_3_. Antibodies for acetyl-histone H3 lysine 27 (#8173), acetylated lysine (#9441), and histone H3 (#4499) were from Cell Signaling Technology. Mouse monoclonal GAPDH (#sc-47724; Santa Cruz Biotechnology) and rabbit monoclonal β-tubulin (#2128; Cell Signaling Technology) were used as a loading control. The acetyl-histone H3 lysine 27 (#8173) antibody was validated in primary human skeletal muscle cells using the histone acetyltransferase inhibitor C646 (Fig 5D). Species-appropriate horseradish peroxidase–conjugated secondary antibodies were used at a concentration of 1:25,000 in 5% skimmed milk in TBS-Tween. Proteins were visualized by chemiluminescence (#RPN2232 ECL and #RPN2235 ECL Select Western Blotting Detection Reagent; GE Healthcare) and quantified using the Image Lab software, v. 5.2.1 (Bio-Rad).

### RNA extraction and RNA sequencing

RNA sequencing from myotube cultures was performed as described (Gabriel et al, 2021). Briefly, RNA was extracted with TRIzol Reagent and miRNeasy kit (Cat #217004; Qiagen) and processed using the Illumina TruSeq Stranded Total RNA with Ribo-Zero Gold protocol (Illumina). Ribosomal RNA was removed, and an RNA sample was fragmented and subjected to first-strand cDNA synthesis. cDNA was subjected to AMPure beads (Beckman Coulter) and adenylated to prime for adapter ligation followed by PCR amplification. Single-end sequencing was performed on the X Ten platform (Illumina) at the Beijing Genomics Institute (BGI). RNA-seq reads ($\bar{n}$ ≈ 38.5 M) from FASTQ files were quality-trimmed using Trim_Galore (v0.4.3) and aligned using STAR (v2.5.3a) (Dobin et al, 2013) with Ensembl human annotation (GRCh38, release 92). Gene features were counted using featureCounts (Liao et al, 2014) from the subread (v1.5.2) package and analyzed with edgeR (Robinson et al, 2010). The logCPM (count per million) values for each gene were calculated using the limma’s *voom* function while correcting for batch effect from participants using the *duplicateCorrelation* function (Ritchie et al, 2015). For the time series data, each gene was normalized using the “scale” function in R. For each gene, data were centered by subtracting the mean value for that gene and scaling was done by dividing the centered data by the SD.

### Acetylated H3K27 chromatin immunoprecipitation and sequencing (ChIP-seq)

Acetylated H3K27 ChIP-sequencing was performed as described (Williams et al, 2020). Myotubes were cross-linked in 1% formaldehyde in PBS for 10 min at room temperature followed by quenching with glycine (0.125 M). Cells were washed with PBS and harvested in 1 ml SDS buffer (50 mM Tris–HCl [pH 8], 100 mM NaCl, 5 mM EDTA [pH 8.0], 0.2% NaN_3_, 0.5% SDS, and 0.5 mM phenylmethylsulfonyl fluoride) and subjected to centrifugation (6 min at 250*g*). Pelleted nuclei were lysed in 1.5 ml ice-cold IP buffer (67 mM Tris–HCl [pH 8], 100 mM NaCl, 5 mM EDTA [pH 8.0], 0.2% NaN_3_, 0.33% SDS, 1.67% Triton X-100, and 0.5 mM phenylmethylsulfonyl fluoride) and sonicated (Diagenode’s Bioruptor) to an average length of 200–500 bp. Before starting the ChIP experiment, chromatin was cleared by centrifugation for 30 min at 20,000*g*. For each ChIP, 2–10 μg DNA was combined with 2.5 μg antibody for H3K27ac (Ab4729) and incubated with rotation at 4°C for 16 h. Immunoprecipitation was performed by incubation with Protein G-Sepharose beads (GE Healthcare) for 4 h followed by three washes with low-salt buffer (20 mM Tris–HCl [pH 8.0], 2 mM EDTA [pH 8.0], 1% Triton X-100, 0.1% SDS, and 150 mM NaCl) and two washes with high-salt buffer (20 mM Tris–HCl [pH 8.0], 2 mM EDTA [pH 8.0], 1% Triton X-100, 0.1% SDS, and 500 mM NaCl). Chromatin was de-cross-linked in 120 μl of 1% SDS and 0.1 M NaHCO_3_ for 6 h at 65°C, and DNA was subsequently purified using the Qiagen MinElute PCR purification kit. For library preparation and sequencing, 3–10 ng of immunoprecipitated DNA was used to generate adapter-ligated DNA libraries using the NEBNext Ultra DNA library kit for Illumina (E7370L; New England Biolabs) and indexed multiplex primers for Illumina sequencing (E7335; New England Biolabs). The PCR cycle number for each library amplification was optimized by running 10% of the library DNA in a real-time PCR using Brilliant III Ultra-Fast SYBR Green qPCR Master Mix (AH Diagnostic) and a C1000 thermal cycler (Bio-Rad). DNA libraries were sequenced on HiSeq 2000 by 50-bp single-end sequencing at the National High-Throughput Sequencing Centre (University of Copenhagen).

### Processing of H3K27ac ChIP-seq data

The quality of H3K27ac ChIP-seq samples was assessed using the FastQC software. One sample was of poor quality and had to be excluded. Quality was trimmed with Trimmomatic (v0.32) (Bolger et al, 2014) on the same parameters to clip adapters to remove low-quality sequences using a minimum quality of 30, a sliding window of 5, and a minimum length of 40 bp. Reads were mapped to *Homo sapiens* reference genome using the Bowtie2 (v2.3.2-foss-2016b) aligner (Langmead & Salzberg, 2012). Duplicate removal was performed using SAMtools (v1.5-foss-2016b) (Li et al, 2009), and then, BAM files were subjected to peak count using the MACS2 software (v2.1.0) (Zhang et al, 2008) for broad peaks using a q-value of 0.01 and a shift of 147 bp. Called peaks were quantified with featureCounts (v1.6.3) (Liao et al, 2014) and associated with closest genes using the RGmatch software (Furio-Tari et al, 2016), mapping regions to the transcriptomic start site and the promoter of the first exon. The resulting peak matrix was Reads Per Kilobase of transcript, per Million mapped reads (RPKM) normalized and batch-corrected using ComBat (Johnson et al, 2007) to correct for sequencing lane bias.

### Bioinformatics analysis

Time series samples were analyzed with the R package RAIN (Thaben & Westermark, 2014) to capture the rhythmic oscillations and determine peak times of the gene expression and acetylated regions. Rhythmicity was determined based on a 24-h longitudinal period. Genes and H3K27-acetylated regions were considered rhythmic when FDR < 0.1. The limma R package (Ritchie et al, 2015) was used to determine differential gene expression and histone acetylation. The effect of time was blocked using a linear function to study the independent effect of palmitate. The effect of palmitate and obesity was considered significant when FDR < 0.1. An enrichment of functional clusters was performed on significant genes (FDR < 0.1) using clusterProfiler (Yu et al, 2012). Results of the analyses are available in Supplemental Data 1.

Supplemental Data 1. Statistical results from the RNA sequencing and H3K27ac-ChIP sequencing experiments.

### Quantitative PCR

Cultured muscle cells were lysed, and RNA was extracted using the E.Z.N.A. Total RNA Kit (Omega Bio-Tek Inc.). All equipment, software, and reagents for performing the reverse transcription and qPCR were from Thermo Fisher Scientific. cDNA synthesis was performed from ∼0.5 μg of RNA using the High Capacity cDNA Reverse Transcription kit (Thermo Fisher Scientific). qPCR was performed on a ViiA7 system with TaqMan Fast Universal PCR Master Mix and predesigned TaqMan probes (Thermo Fisher Scientific).

### Publicly available datasets

The GEO repository was used to download transcriptomic data from biopsies obtained from men before or after a high-fat meal (GSE31901), from men before or after 3 d of a high-fat diet (GSE68231), or from men with obesity versus normal weight (GSE25462, GSE43760, GSE73034, and GSE73078). For the datasets comparing individuals with obesity versus normal weight, only men with either BMI < 25 (healthy) or BMI ≥ 30 (obesity) were selected to enable comparisons with the other studies. In studies assessing the effects of high-fat feeding and obesity, skeletal muscle biopsies were collected after an overnight fast. All studies were processed with similar pipelines using the oligo package in R v4.2.0. Gene ontology pathway enrichment was performed using clusterProfiler (Yu et al, 2012) on the top 2,000 genes in each study, ranked on FDR.

### Statistics

Statistical analyses were performed using the R version 4.2.0. Normality was tested using Shapiro–Wilk’s normality test and equality of variances tested with Levene’s test. The Tukey transformation was used when required to run two-way and three-way ANOVA to determine the overall effect of palmitate, time, and inhibitor in Western blot and qPCR data. An unpaired *t* test was performed to determine the effect of obesity on total histone H3 and histone H3K27ac in skeletal muscle biopsies. *P* < 0.05 was considered significant.

## Data Availability

The raw and processed files for the RNA-seq and the ChIP-seq experiments have been deposited in the GEO repository under accession numbers GSE205424 and GSE205677.

## Supplementary Material

Reviewer comments
